# Importance of Inactivation Methodology in Enzymatic Processing of Raw Potato Starch: NaOCl as Efficient α-Amylase Inactivation Agent

**DOI:** 10.3390/molecules28072947

**Published:** 2023-03-25

**Authors:** Signe Schram Zinck, Stefan Jarl Christensen, Ole Bandsholm Sørensen, Birte Svensson, Anne S. Meyer

**Affiliations:** 1 Department of Biotechnology and Biomedicine, Section for Protein Chemistry and Enzyme Technology, Technical University of Denmark (DTU), Søltofts Plads 224, Kgs., 2800 Lyngby, Denmark; 2 KMC, Herningvej 60, 7330 Brande, Denmark

**Keywords:** α-amylase, enzymatic raw starch modification, potato starch, inactivation methodology, irreversible inactivation, multistep washing, sodium hypochlorite

## Abstract

Efficient inactivation of microbial α-amylases (EC 3.2.1.1) can be a challenge in starch systems as the presence of starch has been shown to enhance the stability of the enzymes. In this study, commonly used inactivation methods, including multistep washing and pH adjustment, were assessed for their efficiency in inactivating different α-amylases in presence of raw potato starch. Furthermore, an effective approach for irreversible α-amylase inactivation using sodium hypochlorite (NaOCl) is demonstrated. Regarding inactivation by extreme pH, the activity of five different α-amylases was either eliminated or significantly reduced at pH 1.5 and 12. However, treatment at extreme pH for 5 min, followed by incubation at pH 6.5, resulted in hydrolysis yields of 42–816% relative to controls that had not been subjected to extreme pH. “Inactivation” by multistep washing with water, ethanol, and acetone followed by gelatinization as preparation for analysis gave significant starch hydrolysis compared to samples inactivated with NaOCl before the wash. This indicates that the further starch degradation observed in samples subjected to washing only took place during the subsequent gelatinization. The current study demonstrates the importance of inactivation methodology in α-amylase-mediated raw starch depolymerization and provides a method for efficient α-amylase inactivation in starch systems.

## 1. Introduction

Potato (*Solanum tuberosum* L.) constitutes one of the most important food staples and is the fourth most cultivated crop worldwide [1]. In addition to direct consumption of the tuber, potatoes and potato-derived products have gained a significant role in the food industry. Today, both native and modified potato starch are widely used as texturizing and stabilizing agents in various processed food products [2]. Native potato starch is comprised of the two major types of α-(1,4)-D-glucose polymers, namely amylose (~18–21%), which is essentially linear, and amylopectin (~79–82%), which contains α-(1,6)-linked branches [1,2]. The main purpose of potato starch modification is to alter its physicochemical properties, so it is tailored to specific applications. Depending on the type of modification, different improved functional properties can be obtained. Traditionally, starch modifications have been carried out by chemical derivatization. Recently, “clean label food” has emerged as a global food trend, reflecting the evolution of consumer preferences for healthier and more sustainable foods. The growing concerns regarding food ingredients and manufacturing have triggered the interest in enzymes as a tool for the production of clean-label modified starches [3,4,5].

The modification of starch at sub-gelatinization temperatures is often preferred to reduce both the cost and environmental impact of manufacturing [6,7,8,9,10]. In raw starch, amylose and amylopectin are packed tightly onto semi-crystalline starch granules of alternating crystalline and amorphous lamellae [11]. The crystallinity of raw starch constitutes the main constraint to enzymatic degradation, and therefore, research focusing on finding, engineering, and assessing effective raw-starch-degrading enzymes for raw starch modification has intensified over the past few decades [9,10,12].

Microbial α-amylases (EC 3.2.1.1) are among the most widely used enzymes in the starch processing industry [12,13]. The enzyme depolymerizes starch by catalyzing the hydrolysis of α-(1,4) linkages of amylose and amylopectin in an endo-manner. This way, α-amylases can be applied to alter the molecular composition of starch, leading to new, unique, and specialized physiochemical properties [5]. The ability to efficiently inactivate the enzyme and thus halt the reaction at a certain level of degradation is a prerequisite for successful controlled enzymatic modification of starch. Contamination with even small amounts of α-amylase can have many adverse effects on the quality and storage stability of starch-containing food. However, the efficient inactivation of α-amylases can be a challenge in starch systems as the presence of the substrate may enhance the structural stability of the enzyme [14,15]. Moreover, the inactivation method has to be chosen with special caution when assessing controlled enzymatic depolymerization of raw starch, as extreme pH or temperature may cause unspecific hydrolysis and/or gelatinization of the starch. The combination of the robustness of α-amylases and the physiochemical nature of raw starch thus restricts the number of possible inactivation methods and makes it particularly difficult to find a method that ensures both rapid, controlled, and irreversible enzyme inactivation.

Some of the most common inactivation methods currently employed in controlled enzymatic raw starch depolymerization are adjustment of pH [16,17,18,19,20,21] and multistep washing with water and/or ethanol [22,23,24,25,26,27,28]. However, notably, multistep washing carries the risk of leaving potentially active α-amylases in the starch, which could lead to residual hydrolytic activity during subsequent processing. Recently, we discovered a patent from 1978 describing a method for differential and *irreversible* inactivation of amylase activity in amylase–protease mixtures using the oxidizing agent, hypochlorite [29]. Hypochlorite may be provided by different hypochlorite salts, such as sodium hypochlorite (NaOCl), commonly known as bleach, which is already one of the most widely used disinfectants in the food industry. Moreover, the substance can easily be reduced to sodium chloride (NaCl) by the addition of sodium bisulfite (NaHSO_3_). Despite its apparent potential, the α-amylase inactivation efficiency of NaOCl does not seem to have been assessed thoroughly.

The objective of this study was to examine the α-amylase inactivation efficiency of pH adjustment, multistep washing, and NaOCl, respectively, in α-amylase-mediated controlled enzymatic raw potato starch depolymerization. Five distinct microbial α-amylases, all belonging to glycoside hydrolase family 13 (GH13) but categorized into different subfamilies according to the CAZy database (http://www.CAZy.org/, 21 February 2023), were employed in the experiments, including three commercial (from Megazyme (Neogen^®^), Wicklow, Ireland) and two non-commercial raw-starch-degrading α-amylases that have previously been described in the literature [30,31] (Table 1). The enzymes will be referred to as *Bl*-αAmy, *Ba*-αAmy and *Ao-*αAmy, *Bt*-αAmy and *Um-*αAmy respectively.

## 2. Results

### 2.1. Inactivation Using pH Adjustment

Raw potato starch samples (15%) were incubated for 16 h with the five different α-amylases, respectively, at pH 1.5 and pH 12 to affirm that none of the enzymes displayed any significant activity at these pH values. As expected, incubation at these extreme pH values resulted in either very low or no detectable hydrolysis yields (Table 2).

However, treatment at extreme pH for 5 min, followed by neutralization and incubation at pH 6.5, resulted in hydrolysis yields of 42–816% compared to the respective controls—i.e., raw potato starch incubated with each of the five α-amylases without preliminary treatment at extreme pH (Table 2). The highest hydrolysis yields were detected when the α-amylases (particularly *Um-*αAmy) were “inactivated” at pH 12 for 5 min, followed by incubation at pH 6.5. It is speculated that these hydrolysis yields >100% might be a result of partial gelatinization of the raw potato starch at pH 12.

### 2.2. Inactivation Using NaOCl

The inactivation efficiency of NaOCl was affirmed by showing that there was no detectable hydrolytic activity in 15% raw potato starch samples incubated for 16 h with each of the five α-amylases in the presence of 7 mM NaOCl (Table 3). Next, the ability of NaOCl to inactivate the α-amylases irreversibly was assessed by treatment with 7 mM NaOCl for 5 min, followed by the reduction of NaOCl with NaHSO_3_ and direct incubation of the samples. The results show that the hydrolytic activity of all five α-amylases on raw potato starch was eliminated after inactivation with 7 mM NaOCl for 5 min followed by reduction with 7 mM NaHSO_3_ (Table 3, +NaOCl+NaHSO_3_), confirming that NaOCl inactivated all five enzymes irreversibly. In the control runs, when incubated with NaHSO_3_ only, a significant amount of enzyme activity was retained (Table 3, −NaOCl+NaHSO_3_). This implies that NaOCl was responsible for the irreversible inactivation of the α-amylases in samples where both reagents were present.

### 2.3. Inactivation by Multistep Washing

**Figure 1:**
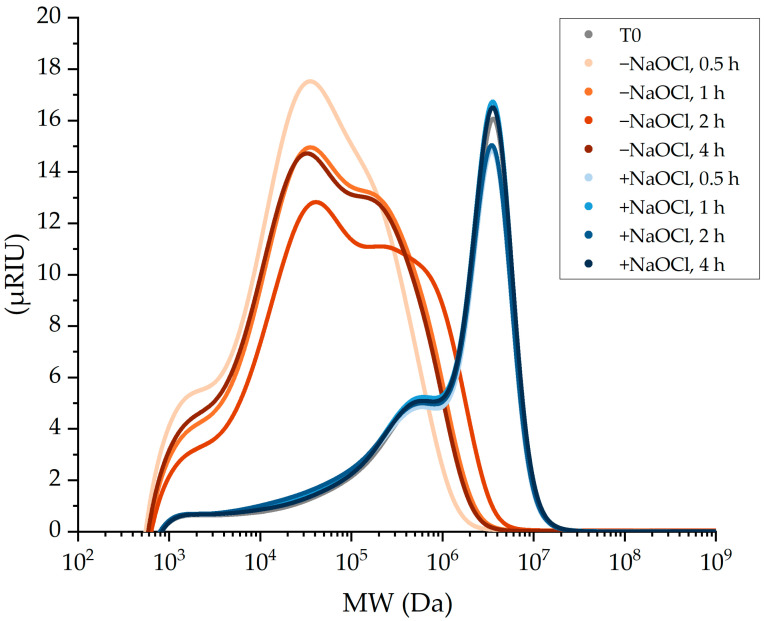
Molecular weight distribution of 15% raw potato starch treated with 11 µg mL^−1^ (~190 nM) Bl-αAmy for 0.5–4 h. Grey chromatogram (T0): native washed potato starch. Orange chromatograms (−NaOCl): samples inactivated by multistep washing only. Blue chromatograms (+NaOCl): samples inactivated with NaOCl (7 mM) before multistep washing.

Inactivation by multistep washing was principally undertaken as described in [22], except that, in the present study, acetone was used in the final washing step. Accordingly, aliquots of 15% raw potato starch treated with 11 µg mL^−1^ *Bl*-αAmy for 0.5, 1, 2 and 4 h were washed with water, ethanol and acetone using a vacuum filter. High-performance size-exclusion (HPSEC) and reducing sugar quantification of the *Bl*-αAmy-treated washed starch samples revealed significant shifts in the molecular weight distribution towards lower molecular weights and higher reducing sugar concentrations, respectively, relative to that of native washed potato starch (Figure 1, orange, and Table 4, respectively).

At first sight, these SEC data appear to indicate that the enzyme caused a significant degree of raw starch degradation. However, the results did not show a consistent and time-dependent reduction of the Mw or reducing sugar increase with increasing incubation time. Hence the suspicion arose that these measurements were confounded by the subsequent gelatinization step required for BCA and HPSEC analysis of the washed- enzyme-treated starch.

To evaluate the enzyme inactivation efficiency of the washing method, the molecular weight distribution and reducing sugar concentration of 11 µg mL^−1^ (~190 nM) *Bl*-αAmy-treated samples, inactivated with NaOCl (7 mM) prior to the washing procedure, were analyzed (Figure 1, blue, and Table 4).

**Figure 2:**
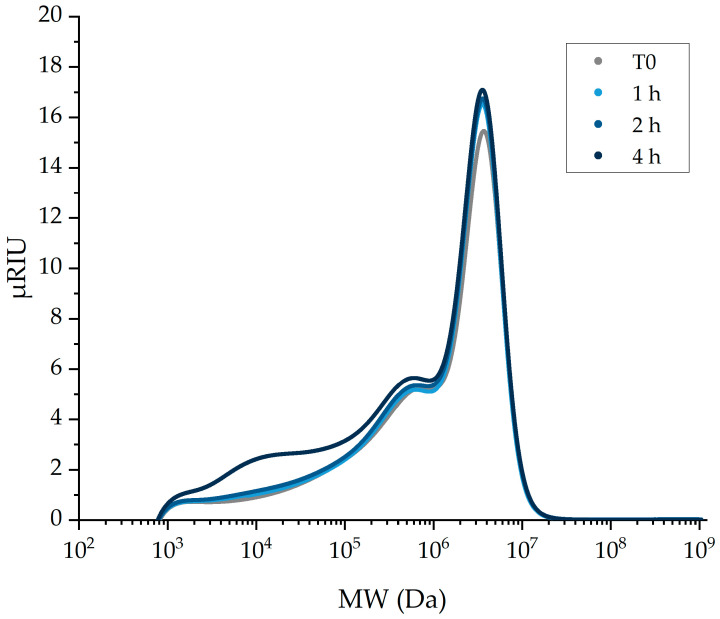
Molecular weight distribution of 15% raw potato starch samples treated with 1.2 mg mL^−1^ (~20 µM) *Bl*-αAmy for 1–4 h and inactivated by 7 mM NaOCl for 5 min before the multistep washing procedure (blue chromatograms). Grey chromatogram (T0): native washed potato starch.

Surprisingly, only minor changes in the molecular weight distribution chromatograms and reducing sugar concentration were obtained when irreversible inactivation of the enzyme was assured. The same was found when the *Bl*-αAmy concentration was 100× higher, i.e., 1.2 mg mL^−1^ (~20 µM) (Figure 2 and Table 5). These results indicate that the multistep washing itself did not inactivate nor remove the enzyme efficiently and confirm that the hydrolytic activity initially observed might be a result of residual activity during the subsequent gelatinization step.

## 3. Discussion and Conclusions

In this study, pH adjustment, NaOCl and multistep washing were tested for their ability to inactivate α-amylase in controlled enzymatic raw potato starch depolymerization.

The loss of hydrolytic activity at pH 1.5 and 12 was more or less reversible for all five α-amylases when pH was neutralized after 5 min. It is suspected that the high pH stability exhibited by the α-amylases in this study is due to the presence of starch, which might prevent irreversible denaturation at extreme pH values by maintaining the structural integrity of the enzyme. The starch may create a microenvironment that allows the α-amylases to retain or re-adopt a native functional conformation, first of all, by shielding the catalytic carboxylic acid residues in the active site from being ionized, and secondly, by promoting non-covalent interactions, such as aromatic pi-stacking and hydrogen bonding, between residues on the surface of the α-amylases and the glucose rings of the substrate [14,15,32]. Because of this, temporarily lowering or increasing the pH, even to extreme values, is not a recommended inactivation method when assessing controlled modification of raw starch using α-amylases. In particular, extremely high pH values are not advisable for enzyme inactivation in raw starch systems, as they may lead to partial gelatinization, and thus, confounded results.

The hydrolytic activity of all five α-amylases on raw potato starch was eliminated both in presence of 7 mM NaOCl during incubation for 16 h and after 5 min treatment with 7 mM NaOCl, confirming irreversible inactivation by this bleaching treatment [29]. The high α-amylase-inactivation efficiency of NaOCl is likely due to it being a powerful oxidant whose ability to damage protein structure has been reported in several studies [33]. A study investigating the action of NaOCl on various free amino acids in different concentration ratios found that when provided in largeexcess, NaOCl reacted very quickly with all of these amino acids, leading to the oxidation and/or formation of chloro derivatives [34]. Based on the results of the current study, NaOCl constitutes an overlooked yet efficient method for fast and complete inactivation of α-amylases, which seems particularly suitable and recommended in controlled enzymatic depolymerization of raw starch where post-reaction gelatinization may confound the results.

**Figure 3:**
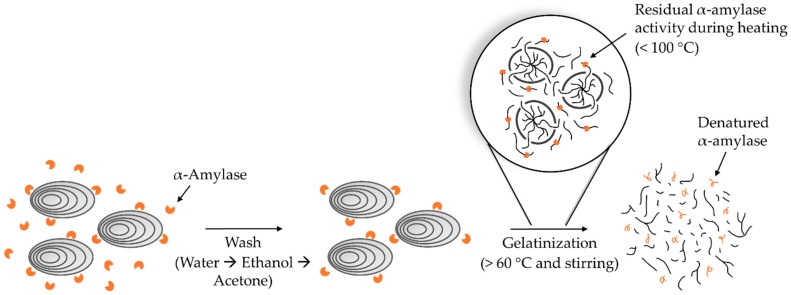
Illustration of how inefficient removal of α-amylase (orange Pac-Man) during the washing steps paves the way for residual hydrolytic activity during subsequent gelatinization of raw potato starch.

A substantial degree of hydrolytic raw starch degradation was observed in *Bl*-αAmy-treated samples “inactivated” by multistep washing only. On the other hand, only minor changes in the molecular weight distribution and reducing sugar concentration were observed in samples inactivated with 7 mM NaOCl prior to the washing procedure. The fluctuating raw starch degradation observed with increasing treatment time when samples were “inactivated” by multistep washing only was likely caused by varying amounts of residual α-amylase being evoked during the subsequent gelatinization step, as illustrated in Figure 3. These results show that multistep washing, even with ethanol and acetone, is not a reliable method to either remove or inactivate α-amylases in controlled raw starch depolymerization.

Even high concentrations of *Bl*-αAmy (~20 µM) caused only minor changes in the molecular weight distribution and reducing sugar concentration of the starch when irreversible inactivation was assured with NaOCl. This indicates that gelatinization might be required for the α-amylase to cause a significant degree of potato starch degradation corresponding to that obtained when samples were subjected to multistep washing only (Figure 1, orange). Raw potato starch is known to be particularly resistant to enzymatic degradation, mainly due to its typical B-type crystallinity. The tight packing of amylose and amylopectin in B-crystalline starch granules creates a smooth granule surface without visible pores that is difficult for an enzyme to attack and penetrate [35,36]. A study by Gérard et al. 2001 [37] found that the extent of α-amylase hydrolysis of raw maize mutant starches was inversely proportional to the amount of B-crystallinity in the starch. It is thus anticipated that the B-type crystallinity of potato starch granules accounts for the limited raw potato starch degradation by *Bl*-Amy, even at high dosages. Therefore, the production of reducing ends observed in samples inactivated properly before gelatinization is most likely a result of the hydrolysis of available starch chains on the granule surfaces (surface erosion).

## 4. Materials and Methods

### 4.1. Materials

Native potato starch (moisture content 19.0% [*w*/*w*, dry basis]; amylose content 20.4 ± 0.6%; purity >99%) was obtained from KMC a.m.b.a. (Brande, Denmark). Commercial α-amylases (E-BLAAM, E-BAASS and E-ANAAM, respectively) were purchased from Megazyme (Wicklow, Ireland). Chemicals were obtained from Merck (Darmstadt, Germany).

### 4.2. Enzyme Production and Purification

The two α-amylase encoding genes were codon-optimized for E. coli expression, synthesized, and cloned into pET28a-TEV in frame with N-terminal His-tag using NdeI and BamHI restriction sites (Genscript, Piscataway, NJ, USA). *E. coli* BL21(DE3) was used as the expression host and routinely propagated in LB broth containing 50 μg mL^−1^ of kanamycin at 37 °C until an OD_600_ of 0.4–0.6 was reached. The cultures were placed on ice for 10 min before the expression was induced by adding 0.5 mM IPTG and 3 mM lactose, followed by expression overnight (~18 h) at 16 °C. Cells were harvested by centrifugation (5500 *g*), sonicated on ice (Qsonica sonicator, Qsonica, Newtown, CT, USA), and the supernatants were collected by centrifugation (19,500 *g*, 4 °C, 20 min). His-tagged proteins were purified as previously described by [38]. Protein purity was verified by SDS-PAGE analysis and protein concentrations were determined spectrophotometrically at 280 nm using theoretical extinction coefficients of 179,510 (Bt-αAmy) and 103,550 M^−1^ cm^−1^ (*Um*-αAmy).

### 4.3. Inactivation Using pH Adjustment

The inactivation efficiency of pH was investigated by measuring the concentration of reducing sugars in duplicate samples of 15% (*w*/*w*) native raw (granular) potato starch incubated (16 h, 40 °C, 1000 rpm) with each of the five α-amylases under different conditions: (1) treatment at pH 1.5 or 12 for 5 min at room temperature, followed by adjustment to pH 6.5 and direct incubation; (2) pH 1.5 or 12 was maintained during incubation; (3) pH was not adjusted (pH 6.5). All pH adjustments were made using NaOH and hydrochloric acid. The following α-amylase concentrations were used: 5.6 µg mL^−1^ (~95 nM) *Bl*-αAmy, 2.3 µg mL^−1^ (~40 nM) *Ba-*αAmy, 8.3 µg mL^−1^ (~152 nM) *Ao-*αAmy, 2.2 µg mL^−1^ (32 nM) *Bt-*αAmy, and 98 µg mL^−1^ (1.3 × 10^3^ nM) *Um-*αAmy. After incubation, the samples were centrifuged at 2000 *g* for 3 min, and the concentration of reducing sugars in the supernatant was measured using bicinchoninic acid assay (BCA) using glucose as standard (Section 4.7).

### 4.4. Inactivation Using NaOCl

The α-amylase-inactivation efficiency of NaOCl was investigated using an experimental setup similar to that described for inactivation by pH adjustment above. The following conditions were applied: (1) treatment with NaOCl for 5 min at room temperature followed by the reduction of NaOCl with NaHSO_3_ (7 mM) and direct incubation (16 h); (2) presence of 7 mM NaOCl during incubation; (3) presence of 7 mM NaHSO_3_ during incubation (4) substrate only. A concentration of 7 mM NaOCl was selected in accordance with the recommended concentration range of 0.2–2% active chlorite [29]. After incubation, the samples were spun down at 2000 *g* for 3 min, and the concentration of reducing sugars in the supernatant was measured using the BCA assay. In addition, the molecular weight distribution (Section 4.6) and reducing sugar concentration were assessed for 1.2 mg mL^−1^ (~20 µM) *Bl*-αAmy-treated raw potato starch inactivated with 7 mM NaOCl before the multistep washing procedure described below.

### 4.5. Inactivation by Multistep Washing

Inactivation by multistep washing was based on the method described by [22], except that an acetone washing step was added, thus conducted as follows: A batch reaction of 100 mL 15% (*w*/*w*) native raw potato starch suspension was placed in a water bath at 40 °C and preheated for 10 min under continuous stirring (500 rpm). The α-amylase treatment was initiated by adding 11 µg mL^−1^ (~190 nM) *Bl*-αAmy. Aliquots (10 mL) of the α-amylase-treated starch suspension were removed at defined time points from 30 min to 4 h and transferred to a Büchner funnel lined with filter paper (mixed cellulose esters membrane, 0.8 µm, Millipore, Darmstadt, Germany) prewashed with water. The supernatant was removed by vacuum filtration, and the starch filtrate residue was sequentially washed three times with ∼100 mL MilliQ water, twice with ~50 mL ethanol (96%, *v*/*v*) and twice with ~50 mL acetone (99.9%, *v*/*v*). The suspensions were mixed thoroughly in each washing step before vacuum filtration. After multistep washing, the treated starch was dried at 30 °C overnight (~18 h), followed by an assessment of molecular weight distribution and quantification of reducing sugars. To evaluate the inactivation efficiency of the multistep wash, the data obtained were compared with those of samples inactivated with 7 mM NaOCl before the washing procedure.

### 4.6. Assessment of Molecular Weight Distribution by High-Performance Size-Exclusion Chromatography

Batches (50 mL) of 1% (*w*/*w*) native and *Bl*-αAmy-treated potato starch were gelatinized according to the previously described method [38]. The samples were diluted to 0.2% (*w*/*v*) in 100 mM acetate buffer, pH 6, and filtered through 0.22 µm nylon filters (Frisenette, Knebel, Denmark). High-Performance Size-Exclusion Chromatography (HPSEC) was performed using an Ultimate iso-3100 SD pump with a WPS-3000 sampler (Thermo Scientific, Waltham, MA, USA) connected to an ERC RefractoMax 520 refractive index detector (Thermo Scientific, Waltham, MA, USA). The column employed was a Shodex SB-806 HQ column (300 × 8 mm) equipped with a Shodex SB-G guard column (50 mm × 6 mm) (Showa Denko K.K., Tokyo, Japan). The samples were run as previously described by [39] using external pullulan standards in the range of 342–805,000 Da (PSS Polymer Standards Service GmbH, Mainz, Germany). Molecular weight above the applied standard range was estimated based on the extrapolation of a third-degree polynomial expression.

### 4.7. Reducing Sugar Quantification by Bicinchoninic Acid Assay

The hydrolytic activity of the α-amylases was determined by quantifying reducing sugars using the bicinchoninic acid (BCA) method [40]. For this analysis, samples of starch supernatant, or 1% gelatinized starch, were diluted 7.5-fold in BCA solution (1.88 mM bicinchoninic acid, 4.0 mM L-serine, 1.66 mM CuSO_4_, 170.6 mM Na_2_CO_3_, and 96.2 mM NaHCO_3_), and incubated at 75 °C for 30 min. The absorbance was measured at 560 nm, and the reducing sugar concentration was determined using glucose as a standard.

## Figures and Tables

**Table 1:** List of the five α-amylases employed in the study.

Enzyme Name	Species	Subfamily	GenBank Accession No.	Product Code (Megazyme)
Commercial α-Amylases				
*Bl*-αAmy	*Bacillus licheniformis*	GH13_5	-	E-BLAAM
*Ba*-αAmy	*Bacillus* *Amyloliquefaciens*	GH13_5	-	E-BAASS
*Ao*-αAmy	*Aspergillus oryzae*	GH13_1	-	E-ANAAM
Non-Commercial α-Amylases				
*Bt-*αAmy	*Bacillus* TS-23	GH13_5	AAA63900.1	-
*Um-*αAmy	Uncultured marine bacterium	GH13_37	AEM89278.1	-

**Table 2:** Inactivation of five distinct α-amylases using pH 1.5 and 12. ^I^ ratio between the reducing sugar concentration in supernatants of 15 % raw potato starch samples incubated for 16 h with each of the five α-amylases at different pH values (S) and the respective controls (C)—i.e., the reducing sugar concentration in supernatants of starch treated with each of the five α-amylase without preliminary treatment at extreme pH—calculated as follows: hydrolysis yield (%) = S/C × 100. ^II^ samples maintained at pH 1.5 or 12 during incubation, ^III^ treatment at pH 1.5 or 12 for 5 min at room temperature, followed by adjustment to pH 6.5 and direct incubation. ND: not detectable. Different superscript letters A,B and a–d in each row and column, respectively, indicate significantly different hydrolysis yields at *p* < 0.05 (one-way ANOVA, based on the Tukey–Kramer statistical comparison test).

Hydrolysis Yield (%) ^I^			
pH	α-Amylase	Incubation at Extreme pH ^II^	Incubation for 5 min at Extreme pH Followed by Incubation at pH 6.5 ^III^
pH 1.5	*Bl*-αAmy	ND ^B^	42.3 ± 5.4 ^A,c^
	*Ba*-αAmy	ND ^B^	66.2 ± 4.7 ^A,b,c^
	*Ao*-αAmy	ND ^B^	75.7 ± 9.6 ^A,a,b^
	*Bt-*αAmy	<0.1 ^B^	99.5 ± 1.5 ^A,a^
	*Um-*αAmy	3.7 ± 0.9 ^B^	69.3 ± 5.8 ^A,b,c^
pH 12	*Bl*-αAmy	0.5 ± 0.1 ^B^	151.2 ± 1.3 ^A,c^
	*Ba*-αAmy	<0.1 ^B^	103.9 ± 2.4 ^A,d^
	*Ao*-αAmy	0.5 ± 0.2 ^B^	251.2 ± 14.7 ^A,b^
	*Bt-*αAmy	<0.1 ^B^	125.9 ± 6.1 ^A,c,d^
	*Um-*αAmy	6.1 ± 0.9 ^B^	815.8 ± 8.1 ^A,a^

**Table 3:** Inactivation test of five α-amylases using 7 mM NaOCl. ^I^ ratio between the reducing sugar concentration in supernatants of 15% raw potato starch samples incubated for 16 h with α-amylase in the presence of NaOCl, NaHSO_3_ or both (S), and the respective controls (C)—i.e., the reducing sugar concentration in supernatants of starch incubated with each of the five α-amylases in the presence of neither NaOCl nor NaHSO_3_—calculated as follows: hydrolysis yield (%) = S/C × 100. ^II^ treatment with 7 mM NaOCl for 5 min at room temperature followed by the reduction of NaOCl with NaHSO_3_ (7 mM) and direct incubation, ^III^ presence of NaOCl during incubation, ^IV^ presence of NaHSO_3_ during incubation. ND: not detectable. Different superscript letters A,B and a–c in each row and column, respectively, indicate significantly different hydrolysis yields at *p* < 0.05 (one-way ANOVA, based on the Tukey–Kramer statistical comparison test).

Hydrolysis Yield (%) ^I^			
α-Amylase	+NaOCl−NaHSO_3_^II^	+NaOCl+NaHSO_3_^III^	−NaOCl+NaHSO_3_^IV^
*Bl*-αAmy	ND ^B^	<<0.1 ^B^	76.8 ± 1.6 ^A,b,c^
*Ba*-αAmy	0.8 ± 0.2 ^B^	ND ^B^	74.8 ± 0.6 ^A,c^
*Ao*-αAmy	ND ^B^	ND ^B^	104 ± 1.7 ^A,a,b^
*Bt-*αAmy	ND ^B^	ND ^B^	96.6 ± 13.6 ^A,b,c^
*Um-*αAmy	ND ^B^	ND ^B^	125 ± 6.6 ^A,a^

**Table 4:** ^I^ Reducing sugar concentration (µM) of 15% raw potato starch treated with 11 µg mL^−1^ (~190 nM) *Bl*-αAmy for 0.5–4 h. ^II^ samples inactivated by multistep washing only. ^III^ samples inactivated by NaOCl (7 mM) before the washing procedure. Different superscript letters A,B and a–d in each row and column, respectively, indicate significantly different reducing sugar concentrations at *p* < 0.05 (one-way ANOVA, based on the Tukey–Kramer statistical comparison test).

Reducing Sugars (µM) ^I^		
Incubation Time (h)	Wash Only ^II^	Inactivation with 7 mM NaOCl for 5 min Prior to Wash ^III^
0.5	477.9 ± 7.2 ^A,a^	10.6 ± 0.9 ^B,c^
1	375.8 ± 10.5 ^A,c^	14.8 ± 1.5 ^B,b^
2	308.7 ± 11.7 ^A,d^	15.9 ± 1.2 ^B,b^
4	428.0 ± 14.5 ^A,b^	17.9 ± 1.2 ^B,a^

**Table 5:** Reducing sugar concentration of 15% raw potato starch treated with 1.2 mg mL^−1^ (~190 nM) *Bl*-αAmy for 1–4 h and inactivated by 7 mM NaOCl for 5 min before the multistep washing procedure. Different superscript letters a–c indicate significantly different reducing sugar concentrations at *p* < 0.05 (one-way ANOVA, based on the Tukey–Kramer statistical comparison test).

Reducing Sugars (µM)	
Incubation Time (h)	Inactivation with 7 mM NaOCl for 5 min Prior to Wash
1	18.7 ± 0.4 ^c^
2	23.3 ± 0.9 ^b^
4	51.4 ± 3.8 ^a^

## Data Availability

All data have been presented in the paper.
